# Bone Marrow Stromal Antigen 2 Is a Novel Plasma Biomarker and Prognosticator for Colorectal Carcinoma: A Secretome-Based Verification Study

**DOI:** 10.1155/2015/874054

**Published:** 2015-10-01

**Authors:** Sum-Fu Chiang, Chih-Yen Kan, Yung-Chin Hsiao, Reiping Tang, Ling-Ling Hsieh, Jy-Ming Chiang, Wen-Sy Tsai, Chien-Yuh Yeh, Pao-Shiu Hsieh, Ying Liang, Jinn-Shiun Chen, Jau-Song Yu

**Affiliations:** ^1^Division of Colon and Rectal Surgery, Chang Gung Memorial Hospital, Linkou, Taiwan; ^2^Graduate Institute of Clinical Medical Sciences, College of Medicine, Chang Gung University, Taoyuan 333, Taiwan; ^3^Department of Cell and Molecular Biology, College of Medicine, Chang Gung University, Taoyuan 333, Taiwan; ^4^Molecular Medicine Research Center, Chang Gung University, Taoyuan 333, Taiwan; ^5^Chang Gung University College of Medicine, Linkou, Taiwan; ^6^Department of Public Health, Chang Gung University, Taoyuan 333, Taiwan; ^7^Pathology Core of the Chang Gung Molecular Medicine Research Center, Taoyuan 333, Taiwan

## Abstract

*Background*. The cancer cell secretome has been recognized as a valuable reservoir for identifying novel serum/plasma biomarkers for different cancers, including colorectal cancer (CRC). This study aimed to verify four CRC cell-secreted proteins (tumor-associated calcium signal transducer 2/trophoblast cell surface antigen 2 (TACSTD2/TROP2), tetraspanin-6 (TSPAN6), bone marrow stromal antigen 2 (BST2), and tumor necrosis factor receptor superfamily member 16 (NGFR)) as potential plasma CRC biomarkers. *Methods*. The study population comprises 152 CRC patients and 152 controls. Target protein levels in plasma and tissue samples were assessed by ELISA and immunohistochemistry, respectively. *Results*. Among the four candidate proteins examined by ELISA in a small sample set, only BST2 showed significantly elevated plasma levels in CRC patients versus controls. Immunohistochemical analysis revealed the overexpression of BST2 in CRC tissues, and higher BST2 expression levels correlated with poorer 5-year survival (46.47% versus 65.57%; *p* = 0.044). Further verification confirmed the elevated plasma BST2 levels in CRC patients (2.35 ± 0.13 ng/mL) versus controls (1.04 ± 0.03 ng/mL) (*p* < 0.01), with an area under the ROC curve (AUC) being 0.858 comparable to that of CEA (0.867). *Conclusion*. BST2, a membrane protein selectively detected in CRC cell secretome, may be a novel plasma biomarker and prognosticator for CRC.

## 1. Introduction

Colorectal cancer (CRC) is the third leading cause of cancer deaths in the United States [1]. In Taiwan, the incidence of CRC increased promptly (up to 70%) during 1991 to 2001. Moreover, CRC had become the third leading cause of cancer death since 1996 and became the first most common malignancy since 2009 in Taiwan. In 2011, there were 14,087 CRC patients diagnosed, and approximately 5,000 patients died of CRC each year. Among them, patients who are younger than 65 years old contributed to about one-third of the CRC death, while the potential life lost was 13.3 years for each patient [2]. Therefore, CRC is an important public health issue and an important socioeconomic problem in Taiwan.

As other malignancies, CRC patients with earlier stage disease have better outcome. In Taiwan, the 5-year survival rate is 81%, 72%, 57%, and 12% for stages I, II, III, and IV diseases, respectively [3]. Furthermore, earlier detection of CRC makes the management more effective and easier. It means earlier diagnosis of CRC can achieve simpler and more straightforward surgery, more cost-effective treatment, better outcome, and better quality of life for patients and somehow avoid post-operative chemotherapy [4–7], although part of stage II patients still suffers disease recurrence [8]. At present, however, definite diagnosis of CRC still depends on colonoscopy. Colonoscopy which is the golden standard of diagnosis of CRC has some disadvantages such as suffering during procedures, risk of colonic perforation and bleeding, and risk of electrolytes imbalance during colon preparation [9–11]. To aid detection and/or monitoring of CRC, carcinoembryonic antigen (CEA), a blood-based tumor marker, has been used extensively in clinic, but it lacks satisfying sensitivity for early tumor [12, 13].

In this “omics” era, the application of high-throughput genomic and proteomic technologies has enabled the discovery of hundreds to thousands of biomarker candidates. However, only very few biomarkers have been brought to clinical settings, and “personalized medicine” is still difficult to achieve [14–16]. The gap between benches to clinics persists. Most previously discovered candidate biomarkers in bench are still lacking rigorous validation, and only few biomarkers had gotten FDA approval in the US. To solve this problem, some researchers questioned sample handling and suggested better study design to decrease selection bias in discovery phase [17–19]. In recent years, more and more strategies have been tried to reduce sample complexity. One of them is proteomic analysis of conditioned media from cancer cell, the so-called cancer cell secretome. The cancer cell secretome-based strategy seems promising in CRC biomarkers discovery. Secreted proteins are easier to be analyzed in cancer cell lines, through which the influence of abundant plasma proteins can be largely reduced. Moreover, cancer cell lines represent a more homogenous cell population than human tissues [20–22]. Comparative analysis of secretomes from different cancer cell lines has been reported. We previously established a 4,584 protein-containing secretomes' dataset of 23 human cancer cell lines from 11 cancer types, in which 109 proteins were selectively identified from three CRC cell lines: Colo205, SW480, and SW620 [21]. These 109 proteins represent a valuable reservoir for further verification study to find novel blood markers for CRC. We integrated these 109 proteins with the Human Protein Atlas (HPA) [23] and Human Plasma Proteome Project (HPPP) [24] datasets and then applied stringent literature search to narrow down candidate list. We have selected four candidates (TACSTD2/TROP2, TM9SF2, TSPAN6, and NGFR) and preliminarily verified their overexpression at protein levels in 30 CRC patients' tissue samples by immunohistochemistry [25]. In the present study, we extended prior work and examined the plasma levels of four targets (TROP2, TSPAN6, NGFR, and BST2) by ELISA of a small sample set of CRC patients and controls and further selected BST2 for detailed analysis. BST2, a type II transmembrane protein also known as HM1.24/CD317, has been identified to be overexpressed in a variety of cell lines from different cancer types, including multiple myeloma, breast, lung, and kidney cancers [26–29]. However, there are still few studies about BST2 expression in human cancers and its potential as a cancer biomarker. Our present study showed that BST2 levels were significantly elevated in both CRC tissues and plasma specimens, implicating the potential of BST2 as a novel CRC biomarker.

## 2. Materials and Methods

### 2.1. Datasets and Criteria for Prioritization of Candidate Biomarkers

Our main dataset comes from secretome of 23 human cancer cell lines derived from 11 cancer cell types, including three CRC cells (Colo 205, SW 480, and SW 620). Among the 4,584 nonredundant proteins identified, there are 109 proteins selectively detected in the CRC cell secretome [21]. These 109 candidates were further prioritized by examining if they (1) are identified in HPPP, (2) are identified to be upregulated over 50% in HPA, (3) are identified to be upregulated in CRC in published references using different laboratory methods, such as microarray, immunohistochemistry (IHC), tissue array, and reverse transcription polymerase chain reaction (RT-PCR), and (4) are secreted proteins or are involved in apoptosis or signal transduction. One exclusion criterion is applied to those which have published ELISA data in CRC research. According to this criterion, 66 proteins were sorted out from the aforementioned 109 CRC-unique candidates into three categories: A, B, and C (see Supplemental Table 1 in Supplementary Material available online at http://dx.doi.org/10.1155/2015/874054). Verifying candidates of categories A and B as clinically useful blood biomarkers for CRC are our prior concern. One protein in category A (TACSTD2/TROP2) and three proteins in category B (TSPAN6, NGFR, and BST2) were selected and verified in the present study owing to available commercial antibodies and more interesting biological functions. TSPAN6 has been reported to be involved in invasive microdomains in cancer cells, which has been found to be involved in tumorigenesis [30, 31]. NGFR has been studied in neurologic malignancy, which is also involved in cell growth control [32–34]. BST2 is a membrane protein which exists as a dimer or a polymer. It was found to stabilize membrane microdomains, to participate in cell adhesion and cell migration, and to block virus budding [35–37].

### 2.2. Patient Population and Clinical Specimens

All clinical samples were collected at Chang Gung Memorial Hospital (Taoyuan, Taiwan). Tissue samples were collected from surgical CRC patients in 1995. Ten CRC tissue specimens were used for initially checking the BST2 protein levels in tumor cells, followed by a large sample set (132 tissue blocks) for more detailed immunohistochemical analysis. Plasma samples were collected from 152 CRC patients before surgery and 152 controls without CRC between 2010 and 2013 according to the protocol as described previously [21]. Briefly, plasma samples were prepared by collecting blood in EDTA tubes (10 mL from each subject) and left at room temperature (for a maximum of 30 min) until centrifugation. Plasma samples were centrifuged at 2,000 ×g for 10 min at room temperature to pellet the cells. After centrifugation, samples were divided into 1.0 mL aliquots in sterile cryotubes and immediately frozen at −80°C for storage until use. All CRC patients had histologically verified adenocarcinoma. Patients' characteristics were obtained from clinical and pathology records including gender, age, tumor location, histological grade, tumor stage, CEA level, preoperative laboratory data, operation date, operation method, tumor recurrence, follow-up date, and follow-up status. All patients were subjected to a follow-up strategy that included regular outpatient visits, CEA test every 3 to 6 months, regular colonoscopy every 1 year to 2 years, and regular image studies (chest X-ray and liver sonography or computed tomography) every year. The characteristics of all study subjects are summarized in Supplemental Table 2. This study was approved by the Institutional Review Board at Chang Gung Memorial Hospital (IRB numbers 99-0515B, 101-0712B, and 102-1446C).

### 2.3. Immunohistochemistry

The tumor tissue blocks used for immunohistochemical staining were first fixed in 4% paraformaldehyde and then embedded in paraffin. The paraffin embedded tumor sections (5 *μ*m) were deparaffinized with xylene, dehydrated with ethanol, heated in citrate buffer, and then exposed to 3% H_2_O_2_ at room temperature for 30 min before heating in a microwave oven for antigen retrieval (10 mm citrate buffer, pH 6.0; 20 min, 700 W). The sections were blocked with 10% nonimmune goat serum at 37°C for 30 min. Slides were then incubated with rabbit anti-human BST2 antibody (catalog number HPA017060; Sigma-Aldrich, St. Louis, MO) for 30 min at room temperature. Following washing with PBS (pH 7.4), slides were incubated with HRP-conjugated anti-rabbit IgG antibody (1 : 2000 dilution; Abcam, Inc., Cambridge, UK) for 30 min at room temperature and then developed using 3,3′-diaminobenzidine (Sigma, St. Louis, MO). Sections were counterstained with hematoxylin, washed in running tap water, dehydrated, and mounted in Neo-Mount (Merck, Darmstadt, Germany). Immunostaining was evaluated and scored by two experienced pathologists who were blinded to any knowledge of clinical or pathological parameters and clinical outcome. The percentage of antigen-positive tumor cells was determined semiquantitatively by assessing the entire tumor section. Expression of these protein was categorized as positive or negative and was evaluated according to the percentage of cells stained (0–100%) and the intensity of cell staining (3: strong; 2: moderate; 1: weak; or 0: no cell staining). The two scores are multiplied to obtain the final score.

### 2.4. ELISA of TSPAN6, BST2, NGFR, and CEA

Commercial ELISA kits were used for three candidates (Human TSPAN6 ELISA kit, Cusabio Cat#: CSB-EL025164HU; Human BST2 ELISA kit, Cusabio Cat#: CSB-EL002837HU; Human NGFR ELISA kit, RayBio Cat#: ELH-NGFR-001) as below. Briefly, 100 *μ*L per well of standard and sample was added on 96-well coated plates and was incubated at 37°C for 2 hours (overnight at 4°C for NGFR). After removing the liquid, 100 *μ*L of biotin-antibody was added to each well and incubated at 37°C for 1 hour. After adequate aspiration and washing, 100 *μ*L of HRP-avidin was added to each well and incubated at 37°C for 1 hour. Repeat the aspiration/wash process, and add 90 *μ*L of TMB substrate. Incubate for 15–30 minutes under light protection at 37°C. Finally, add 50 *μ*L of stop solution. Then, determine the optical density within 5 minutes. Subtract readings at 540 nm or 570 nm from the readings at 450 nm. The procedures all followed manufacturers' protocol. The CEA concentrations in plasma samples were measured using a commercial ELISA kit of CEA (Carcinoembryonic Antigen ELISA, BQ Kits Cat#: BQ062T). Fifty microliters per well of standard and sample was added on 96-well coated plates. Then, 100 *μ*L of CEA enzyme conjugate was added to all wells and incubated at room temperature for 1 hour. After the aspiration/wash process, 100 *μ*L of TMB substrate was added to each well and incubated at room temperature for 10 minutes. After adding 50 *μ*L of stop solution to each well, the absorbance at 450 nm was recorded on ELISA Reader within 15 minutes.

### 2.5. Sandwich ELISA of TROP2 (TACSTD2)

In-house ELISA of TROP2 was developed as previously described [38]. White polystyrene 96-well microtiter plates (Corning, NY, USA) were coated with goat anti-TROP2 antibodies (AF650, R&D Systems, Minneapolis, MN) by incubation at 4000 ng/mL in PBS (50 *μ*L in each well) for 2 hours at room temperature. After washing, the plates were blocked by the addition of 200 *μ*L per well of 1% BSA (Sigma)/PBS and incubated overnight at 4°C. After washing with PBS, 50 *μ*L of plasma sample diluted 1 : 10 in blocking buffer was added and incubated at room temperature for 1 hour. Recombinant TROP2 protein (650-T2, R&D) was used as a standard. Biotinylated anti-human TROP2 (BAF650, R&D) antibodies (1 : 50 dilution in PBS containing 1% BSA) were applied and the plates were incubated at room temperature for 1 hour. Then, the streptavidin-alkaline phosphatase (RPN1234, Amersham Bioscience, UK) (50 *μ*L, diluted 3000-fold in PBS containing 1% BSA) was added and incubated at room temperature for 40 min. One hundred microliters of substrate 4-methylumbelliferyl phosphate (Molecular Probes, Eugene, OR) (diluted to 100 *μ*M with alkaline phosphatase buffer) was added to each well. The fluorescence was measured with a SpectraMax M5 microplate reader (Molecular Devices, Sunnyvale, CA, USA) with excitation and emission wavelength set at 355 and 460 nm, respectively.

### 2.6. Statistical Analysis

The relationship between clinicopathologic features and BST2 protein expression levels was assessed by the chi-square method. Mean values of BST2 protein expression in different groups were compared by independent *t*-test or ANOVA method. Overall survival and time-to-event probabilities were computed using univariate analysis by the Kaplan-Meier method. Differences were estimated by log-rank test. Receiver operating characteristic (ROC) analysis was performed for plasma BST2 and CEA in discriminating CRC patients from controls. Multivariate analysis was done using Cox proportional hazard models. Statistical significance was set at *p* < 0.05. All analyses were performed using the statistical software, Statistical Package for the Social Sciences (Version 13.0, SPSS Inc., Chicago, IL).

## 3. Results

### 3.1. Initial Measurement of Plasma Levels of Four Candidates (TROP2, TSPAN6, BST2, and NGFR) in a Small Sample Set

**Figure 1:**
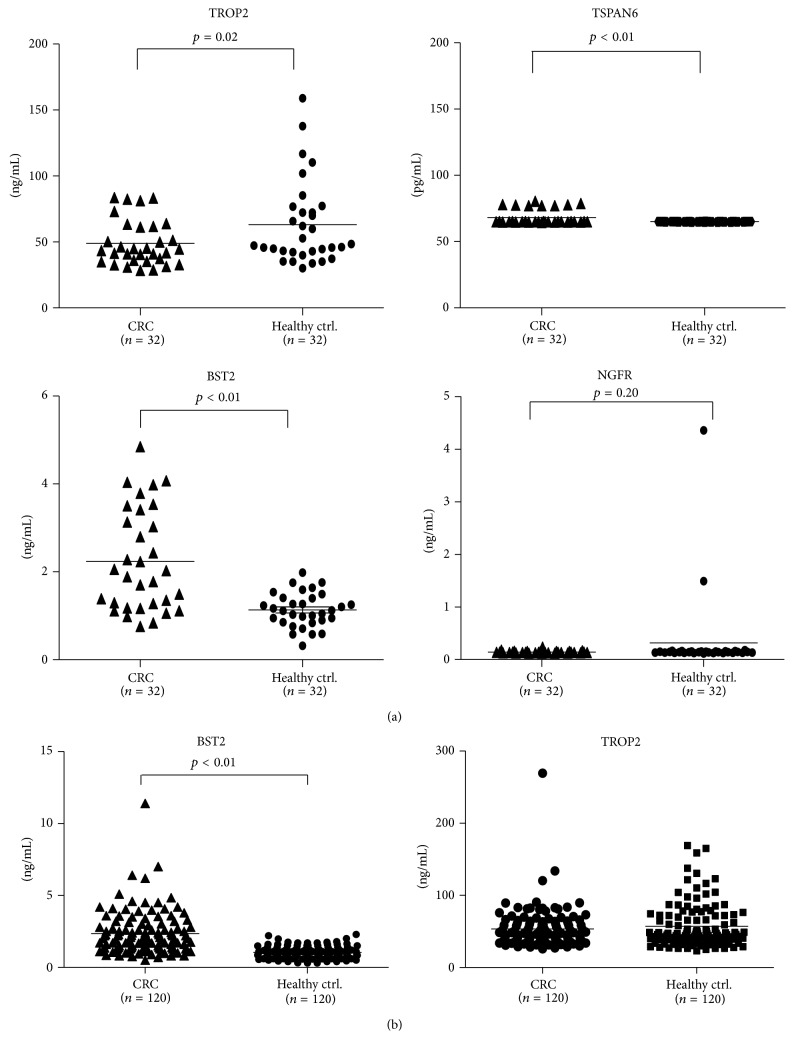
ELISA for four candidate proteins in plasma samples from CRC patients and healthy controls. (a) Plasma samples from 32 CRC patients and 32 healthy controls were used in this study. All *p* values are shown on figures. (b) Extended verification of ELISA of BST2 and TROP2 in another plasma sample set containing 120 CRC patients and 120 healthy controls.

Four candidate proteins, including TROP2, TSPAN6, BST2, and NGFR, were selected for initial verification by ELISA in plasma samples from 32 CRC patients and 32 healthy controls. The plasma levels of TROP2, TSPAN6, BST2, and NGFR in CRC patients and healthy controls were determined to be 48.88 ± 3.00 ng/mL versus 63.05 ± 5.61 ng/mL (*p* = 0.02), 68.15 ± 1.02 pg/mL versus 65.02 ± 0.01 pg/mL (*p* < 0.01), 2.23 ± 0.20 ng/mL versus 1.13 ± 0.06 ng/mL (*p* < 0.01), and 140.00 ± 3.85 pg/mL versus 314.40 ± 137.00 pg/mL (*p* = 0.20), respectively (Figure 1(a)). This analysis suggests that the plasma levels of TROP2 and BST2 might have been significantly altered in CRC patients, which deserves further verification in a large sample set.

### 3.2. Extended Verification of Plasma TROP2 and BST2 Levels in a Large Sample Set

We then performed extended verification of TROP2 and BST2 in another independent plasma sample set, consisting of 120 CRC patients and 120 controls. In agreement with the previous result, the plasma BST2 levels still showed a significant increase in CRC patients as compared to the controls in this independent sample set (2.35 ± 0.13 ng/mL versus 1.04 ± 0.03 ng/mL, *p* < 0.01, independent *t*-test; Figure 1(b),* left panel*). Under the same assay condition, however, plasma TROP2 levels did not maintain significant difference between CRC patients and controls (53.37 ± 12.27 ng/mL versus 57.11 ± 15.02 ng/mL; *p* = 0.31, independent *t*-test; Figure 1(b),* right panel*). We further examined the relationship between plasma BST2 levels and different clinicopathologic characteristics of these 120 CRC patients. Although plasma BST2 levels did not significantly differ in CRC patients at different stages (Supplemental Figure 1), higher plasma BST2 levels were observed in older patients (2.61 ± 1.34 versus 2.03 ± 1.63; *p* = 0.03), mucinous carcinoma (4.63 ± 0.45 versus 2.28 ± 1.48; *p* = 0.05), and CRC patients with hypoalbuminemia (4.14 ± 2.46 versus 2.12 ± 1.14; *p* < 0.01) (Supplemental Table 3).

### 3.3. Overexpression of BST2 in Tumor Cells of CRC Tissues

**Figure 2:**
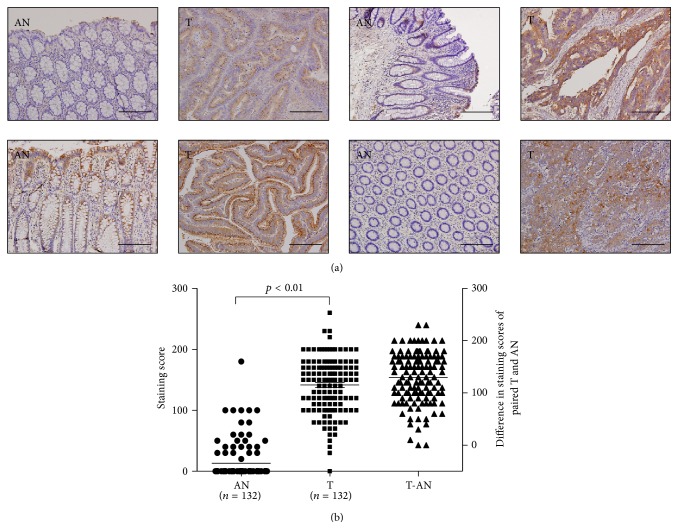
Overexpression of BST2 in CRC tissues. (a) The representative pictures of immunohistochemical staining patterns of BST2 in four pairs of tumor (T) and adjacent normal counterpart (AN) sections (scale bar = 200 *μ*m). (b) Analysis of the IHC scores of BST2 in 132 CRC tissue specimens harboring both tumor and adjacent normal counterpart. T-AN: the difference of IHC score between paired T and AN.

Since we have observed the significant elevation of BST2 plasma levels in CRC patients, we then turned to examine the expression levels of BST2 in CRC tissue specimens by immunohistochemistry. Although BST2 has been identified to be overexpressed in a variety of cell lines from different cancer types [26–29], to our knowledge, there were no studies reporting BST2 protein expression in CRC tissue specimens. In the majority of 132 CRC tissue specimens examined and evaluated, the BST2 antibody strongly stained the cytoplasm of tumor cells but stained weakly or not at all the adjacent nontumor epithelial cells (see Figure 2(a) for representative images). The immunohistochemical staining (IHC) scores of tumor parts were found to be significantly higher than those of adjacent nontumor counterparts (141.60 ± 45.14 versus 13.14 ± 3.00, *p* < 0.01, independent *t*-test; Figure 2(b)). We further examined the relationship between BST2 tissue expression levels and different clinicopathologic characteristics of these 132 CRC patients. We found that higher BST2 tissue expression levels were associated with higher TNM stages and worse 5-year survival, respectively (Supplemental Table 4). The BST2 tissue expression levels were not significantly associated with other clinicopathologic characteristics, such as gender, age, tumor location, histological grade, and distant metastasis.

### 3.4. Tissue BST2 Levels and Overall Survival

**Figure 3:**
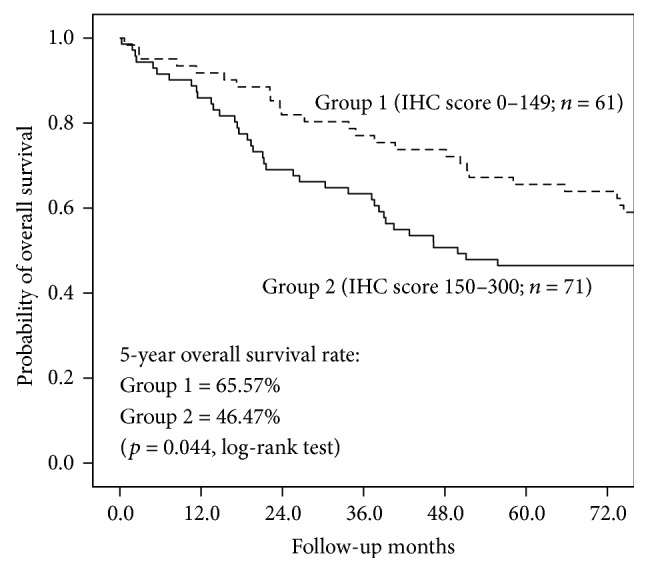
Association of BST2 tissue expression levels with survival among CRC patients used in this study. The IHC score of 150 of BST2 was used as cutoff value for survival analysis of 132 CRC patients.

The 132 CRC patients were stratified into two groups representing high versus low BST2 expression. An IHC score of 150 was selected as cutoff value because (i) this score is the median of the IHC scores of these 132 CRC tissue sections, which can divide these CRC cases into subgroups with comparable sizes, and (ii) most of the adjacent nontumor counterparts have IHC scores below 150. Overall survival of these CRC patients and their time-to-event probabilities were computed using univariate analysis by the Kaplan-Meier method. The result showed that CRC patients of group 1 (IHC score 0–149, *n* = 61) had 5-year survival rate of 65.57%, better than 46.47% of group 2 (IHC score 150–300, *n* = 71; *p* = 0.044, log-rank test; Figure 3), indicating BST2 tissue expression level as a potential prognostic factor of CRC patients.

### 3.5. Multivariate Analysis

In multivariate analysis, BST2 tissue expression (IHC score) still showed marginal effect on 5-year survival (Table 1). The hazard ratios of high protein expression compared to low expression were 1.64 (95% CI, 0.98–2.74, *p* = 0.05). BST2 showed significance with marginal *p* value in multivariate analysis.

### 3.6. ROC Analysis of BST2 and CEA

**Figure 4:**
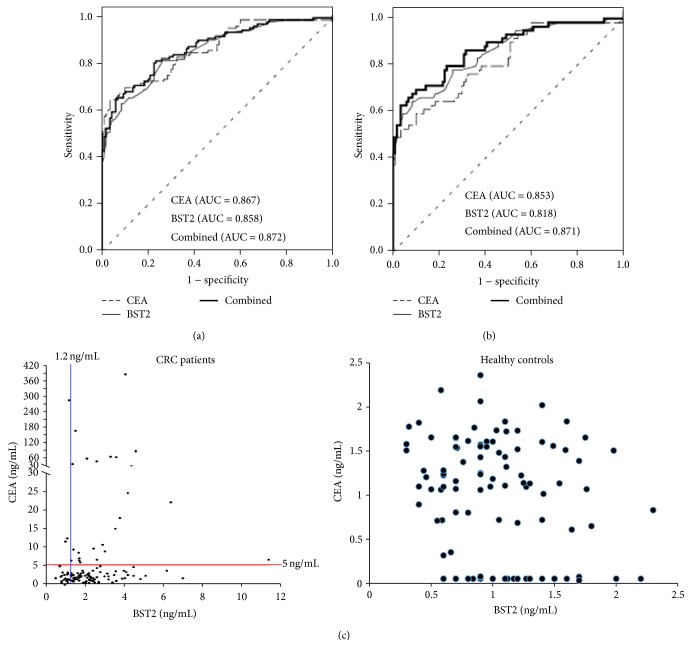
ROC curve analyses for the use of BST2, CEA, and the two-marker panel in discriminating CRC patients from healthy controls. (a) Analysis using all stage CRC patients (*n* = 120) and healthy controls (*n* = 120). (b) Analysis using stages I-II CRC patients (*n* = 61) from healthy controls (*n* = 120). (c) The distributions of plasma levels of BST2 and CEA among the 120 CRC patients and 120 healthy controls (CEA-BST2 plot).

We performed ROC analysis to evaluate the efficacy of plasma BST2 and CEA levels for discriminating CRC patients (*n* = 120) and controls (*n* = 120). The area under the ROC curve (AUC) was 0.858 (95% CI, 0.811–0.904) for BST2, 0.867 (95% CI, 0.821–0.912) for CEA, and 0.872 (95% CI, 0.828–0.916) for combination of BST2 and CEA (Figure 4(a)). We did the same analysis for early stage (stage 1 to stage 2) CRC patients (*n* = 61) and controls. The AUC was determined to be 0.818 (95% CI, 0.751–0.886) for BST2, 0.853 (95% CI, 0.792–0.914) for CEA, and 0.871 (95% CI, 0.813–0.929) for combination of BST2 and CEA (Figure 4(b)). Furthermore, when a cutoff value of 5.0 ng/mL was chosen for CEA as clinical practice and applied to the sample set used here (120 CRC patients and 120 controls), the sensitivity was 23.5% and the specificity was 100.0%. Notably, when a cutoff value of 1.20 ng/mL was chosen for BST2 (with 81.7% sensitivity and 64.2% specificity), 74 of 93 CRC patients with CEA level lower than 5.0 ng/mL could be further distinguished from healthy controls (Figure 4(c)). Taken together, these results indicate that BST2 represents a potential, novel plasma biomarker for CRC, especially when used together with CEA.

## 4. Discussion

CRC is an important public health issue and a socioeconomic problem in Taiwan. Earlier detection of CRC makes better outcome and simpler treatment. Metastasized disease has much worse outcome than localized disease [39]. At present, colonoscopy is still the golden standard of diagnosis of CRC. However, the potential severe complication of colonoscopy should be warranted. The rate of major complications has been reported at 0.12% for perforation and 0.2% for bleeding [40, 41]. Carcinoembryonic antigen (CEA), discovered by Gold and Freedman in the 1960s, is now the only biomarker used for CRC clinically [42]. As a blood biomarker of CRC, the specificity and sensitivity of CEA are around 70~80% [12, 43]. The sensitivity of CEA for early colon cancer patients is low and increases with an increasing stage of the disease. The sensitivity in stages I~II, stage III, and stage IV CRC is 36%, 74%, and 83%, respectively [44]. Therefore, the false negative rates are relatively high for both diagnosis and detection of metastasis.

Although hundreds to thousands of biomarker candidates had been found through the applications of advanced high-throughput genomic and/or proteomic technologies, only very few among them have been verified and brought to clinical settings [15, 16, 45]. In contrast to the extensive survey on genomics and transcriptomes of human CRC, the proteomics survey in CRC is still limited. Currently, only few CRC biomarkers had been approved by FDA. They are CEA, fibrin/fibrinogen degradation product (DR-70), and human hemoglobin (fecal occult blood) [46]. Until now, there is still no single CRC biomarker comparable to CEA.

The present study applying secretome-based strategy has successfully verified BST2 as a potential CRC plasma biomarker for the first time. BST2 (also known as tetherin/CD137/HM1.24 antigen) is a transmembrane glycoprotein with a molecular weight of 19.7 kDa. The gene encoding BST2 was initially cloned in 1995 and reported to be involved in pre-B cell growth via cell-cell interaction [25, 47]. In 2008, BST2 was identified as a restrictive factor of HIV-1 replication [48]. BST2 functions as the physical link between HIV-1 particles and retains virion particles in restrictive cells, and it is responsive to interferon [49]. In recent years, several studies reported the connection between BST2 and cancer. In 2009, Cai et al. reported that BST2 protein expression is associated with bone metastasis in human breast cancer. They found overexpression of BST2 in the bone metastatic breast cancer tissues (compared to nonbone metastatic breast cancer tissues), as well as elevated BST2 levels in breast cancer patients with bone metastasis (compared to breast cancer patients without bone metastasis) [27]. In 2013, Yokoyama et al. reported the overexpression of BST2 in endometrial cancer and found the cytotoxic effect of anti-BST2 antibody on endometrial cancer cells in vitro and in an in vivo xenograft model [50]. More recently, Fang et al. showed that overexpression of BST2 is associated with nodal metastasis and poorer prognosis in oral cavity cancer [51]. Our present study demonstrated the overexpression of BST2 in tumor cells of CRC tissues, which is correlated with poor prognosis of CRC patients. We also showed the significant elevation of plasma BST2 levels in CRC patients compared to normal controls and showed that BST2 may increase AUC after combining CEA for CRC detection, especially for stage 1-2 CRC detection (Figure 4). However, unlike CEA, plasma BST2 levels did not correlate with the disease progression of CRC in the sample set used in this study (Supplemental Figure 1). As an unusual type II transmembrane protein found in lipid rafts, the mechanistic involvement of BST2 in malignancies is not clear yet.

Although our data showed the significant elevation of tissue and plasma BST2 levels in CRC patients compared to normal controls, how BST2, a type II transmembrane protein, can be released from tumor tissue into the blood circulation remains unclear at present. According to the information from Ensembl database, three BST2 transcripts have been identified to date; only one of them represents protein coding transcript and the other two are processed, nonprotein coding transcripts [52]. As a type II transmembrane protein, BST2 has been shown to be involved in microdomains of cell membrane [35, 53]. Several previous studies regarding the identification of proteins in exosomes derived from a variety of cell/tissue types provided important clue about the potential mechanism for shedding of BST2 into blood circulation. Using mass spectrometry-based proteomics approach, BST2 has been repeatedly detected in the exosomal fractions purified from B cells [54], ovarian cancer cells [55], thymic tissues [56], and urine [57]. Taken together, these observations suggest that the exosome-based secretion pathway may represent one of the potential mechanisms for shedding of BST2 into blood circulation from CRC tumor cells. This obviously represents an intriguing question that deserves further investigation.

In conclusion, we found a marked difference of BST2, a membrane protein selectively detected in CRC cell secretome, between CRC patients and healthy controls. The combination of BST2 and CEA outperformed each marker alone in distinguishing CRC patients from healthy individuals. The plasma level of BST2 may be a potential novel CRC biomarker.

## Supplementary Material

Supplementary materials provide some extra information about category setting, clinicopathologic features of CRC patients, and mean plasma level of BST2 according to tumor stage. BST2 did not show its defference among different stages. These materials were not included in the maintext, however, they were still important in our research. Supplementary Table 1 showed our priority of candidates validation. Six criterias were used, including two large databases, HPA and HPPP. Secretome based verification is a tremendous work, and these supplementary materials showed details of our strategy.

## Figures and Tables

**Table 1:** Multivariate analysis of BST2 tissue expression levels and clinicopathologic factors of 132 CRC patients.

Variable	HR^*∗*^	95% CI^*∗∗*^	*p* value
Gender			
Male	1		
Female	0.70	0.42~1.17	0.17
Age (years)			
<65	1		
≧65	1.91	1.18~3.11	<0.01
TNM stage			
Early (I~II)	1		
Late (III~IV)	3.29	1.94~5.58	<0.01
Differentiation			
Well	1		
Moderate	1.25	0.66~2.36	0.01
Poor	1.77	0.55~5.69	
Histological type			
Adenocarcinoma	1		
Mucinous carcinoma	1.82	0.65~5.14	0.25
Chemotherapy			
Yes	1		
No	1.40	0.80~2.46	0.23
CEA (ng/mL)			
<5	1		
≧5	2.02	1.21~3.37	<0.01
BST2 (IHC score)			
Low (0–149)	1		
High (150–300)	1.64	0.98~2.74	0.05

^*∗*^HR: hazard ratio.

^*∗∗*^CI: confidence interval.
