# Supplementary material for: Bone Marrow Stromal Antigen 2 Is a Novel Plasma Biomarker and Prognosticator for Colorectal Carcinoma: A Secretome-Based Verification Study
Source: Dis Markers. 2015 Oct 1;2015:874054. doi: 10.1155/2015/874054 (PMC4606116; doi:10.1155/2015/874054)
Supplement: Supplementary file 1 — Supplementary materials provide some extra information about category setting, clinicopathologic features of CRC patients, and mean plasma level of BST2 according to tumor stage. BST2 did not show its defference among different stages. These materials were not included in the maintext, however, they were still important in our research. Supplementary Table 1 showed our priority of candidates validation. Six criterias were used, including two large databases, HPA and HPPP. Secretome based verification is a tremendous work, and these supplementary materials showed details of our strategy. [file 874054.f1.pdf]

Supplemental Table 1. Prioritization and selection of candidate biomarkers from CRC cell secretomes.

Category	Criteria*	Protein name	Gene symbol	Criterion 1	Criterion 2	Criterion 3	Criterion 4	Criterion 5
A	1+2 or 1+3	Tumor-associated calcium signal transducer 2/trophoblast cell surface antigen 2	TACSTD2/TROP2**	Yes	No	Ref. 1~6		No
		Transmembrane 9 superfamily member 2	TM9SF2	Yes	Yes			No
B	1+4 or 2+3 or 2+4	Tetraspanin-6	TSPAN6	No	Yes		Ref. 7~9	No
		Bone marrow stromal antigen 2	BST2	No	Yes	Ref. 10~13		Yes
		Tumor necrosis factor receptor superfamily member 16	NGFR	No	Yes	Ref. 14~18		No
		Glia-activating factor	FGF9	No	Yes	Ref. 19~23		No
		Isoform 3 of Canalicular multispecific organic anion transporter 2	ABCC3***	Yes	No	Ref. 24~28		No
		Cell surface A33 antigen	GPA33	No	Yes	Ref. 29~33		No
		55 kDa erythrocyte membrane protein	MPP1	No	Yes		Ref. 34~37	No
		142 kDa protein	PCDH24	No	Yes	Ref. 38		No
		Isoform 2 of Ankyrin repeat-rich membrane spanning protein	KIDINS220	No	Yes	Ref. 39		No
		Flotillin 2	FLOT2	No	Yes	Ref. 40~43		No
		Trefoil factor 3	TFF3	No	Yes	Ref. 44~48		Yes
		Isoform long of Antigen KI-67	MKI67	No	Yes	Ref. 49~52		No
		22 kDa protein	TIMP2	No	Yes	Ref. 53~56		No
		Claudin-3	CLDN3	No	Yes	Ref. 57~62		No
		Metallothionein-3	MT3	No	Yes	Ref. 63~66		No
C	2 or 3 or 4	EPHB6 protein	EPHB6	No	No	Ref. 67~71		No
		Rho-related BTB domain-containing protein 3	RHOBTB3	No	Yes			No
		Galactoside 3(4)-L-fucosyltransferase	FUT3	No	No	Ref. 72~74		No
		Protein VAC14 homolog	VAC14	No	Yes			No
		Isoform 1 of TRAF2 and NCK-interacting protein kinase	TNIK	No	Yes			No
		Isoform 3 of Misshapen-like kinase 1	MINK1	No	No		Ref. 75~77	No
		Protein-tyrosine kinase fyn isoform c	FYN	No	Yes			No
		Isoform 2 of protein phosphatase slingshot homolog 3	SSH3	No	Yes			No
		Xylosyltransferase 1	XYLT1****	Yes	Yes			No
		70 kDa protein	SLC6A6	No	Yes			No
		Protein APCDD1	APCDD1	No	Yes			No
		Isoform CSBP1 of Mitogen-activated protein kinase 14	MAPK14	No	Yes			No
		Ephrin type-B receptor 3	EPHB3	No	Yes			No
		Ataxia telangiectasia mutated protein isoform 2	ATM	No	Yes			No
		Stromal cell-derived factor 2	SDF2	No	No	Ref. 78~79		No
		Perforin-1	PRF1	No	No	Ref. 80		No
		Inhibin beta B chain	INHBB	No	No	Ref. 81~84		No
		Coagulation factor XIII A chain	F13A1	No	No	Ref. 85~86		Yes
		Low-density lipoprotein receptor-related protein 4	LRP4	No	Yes			No
		CDC42 binding protein kinase alpha	CDC42BPA	No	No	Ref. 87		No
		Isoform long of Glycylpeptide N-tetradecanoyltransferase 1	NMT1	No	Yes			No
		Cell growth regulator with EF hand domain protein 1	CGREF1	No	Yes			Yes
		Isoform 1 of protein KIAA1199	KIAA1199	No	No	Ref. 88~90		No
		AP1B1 protein	AP1B1	No	No		Ref. 91~94	No
		Ladinin 1	LAD1	No	Yes			No
		Isoform 1 of von Willebrand factor A domain-containing protein 2	VWA2	No	No	Ref. 95		Yes
		Isoform 1 of Transmembrane protein 16A	TMEM16A	No	No	Ref. 96~98		No
		Isoform 2 of Condensin-2 complex subunit G2	NCAPG2	No	Yes			No
		Prostatic acid phosphatase	ACPP	No	Yes			No
		9 kDa protein	RPS27	No	No	Ref. 99~102		No
		Isoform 2 of General transcription factor 3C polypeptide 5	GTF3C5	No	No	Ref. 103~104		No
		Isoform 2 of Transmembrane and TPR repeat-containing protein 3	TMTC3	No	Yes			No
		Catenin, beta like 1	CTNNBL1	No	Yes			No

Ras GTPase-activating-like protein IQGAP3	IQGAP3	No	No	Ref. 105~107		Yes
Isoform 1 of PDZ domain-containing protein 11	PDZD11	No	Yes			No
Lipocalin 2	LCN2	No	Yes			No
cDNA FLJ46245 fis, clone TESTI4020596, highly similar to Homo sapiens calpain 5	CAPN5	No	No	Ref. 108~109		No
Proliferating-cell nucleolar antigen p120	NOL1	No	No	Ref. 110~112		No
Isoform 1 of Pregnancy-specific beta-1-glycoprotein 11	PSG11	No	No	Ref. 113~115		No
Isoform 1 of Choline transporter-like protein 1	SLC44A1	No	No	Ref. 116		No
Phosphoglycerate mutase 2	PGAM2	No	No	Ref. 117~118		Yes
Isoform A of Nucleoporin SEH1-like	SEH1L	No	Yes			No
Fibroblast growth factor 19	FGF19	No	No	Ref. 119~120		No
Isoform 2 of Chloride intracellular channel protein 5	CLIC5	No	No	Ref. 121		No
36 kDa protein	MFNG	No	No		Ref. 122	No
Eukaryotic translation initiation factor 1A, Y-chromosomal	EIF1AY	No	Yes			Yes
Calcium and integrin-binding protein 1	CIB1	No	Yes			No
Neutral amino acid transporter A	SLC1A4	No	Yes			No
Receptor-type tyrosine-protein phosphatase epsilon precursor	PTPRE	No	Yes			No

* Criteria:
(1) proteins detected in the high-confidence human plasma proteome reference set established in 2006 [States et al., 2006]
(2) proteins overexpressed in CRC tissue specimens in the Human Protein Atlas (HPA) dataset [Bjorling et al., 2008]
(3) proteins up-regulated in CRC in published references
(4) functions as secreted proteins, or involving in apoptosis/signal transduction
(5) proteins detected in the high-confidence human plasma proteome reference set established in 2011 [Farrah et al., 2011]
(6) proteins overexpressed in CRC tissue speciemens in the Human Protein Atlas (HPA) dataset, Protein Atlas version 13 - 2014.11.06
** TACSTD2(TROP2) was enrolled in Protein Atlas version 12 - 2013.12.05
*** ABCC3 was set in category B due to previous literature lacking positive association in CRC.
**** XYLT1 was set in category C because it is an enzyme which was not a favorable candidate for cancer biomarker.

References:
1 Identification of Trop-2 as an oncogene and an attractive therapeutic target in colon cancers. Wang J, Day R, Dong Y, Weintraub SJ, Michel L. Mol Cancer T
2 Clinical significance of TROP2 expression in colorectal cancer. Ohmachi T, Tanaka F, Mimori K, Inoue H, Yanaga K, Mori M. Clin Cancer Res. 2006 May
3 Overexpression of TROP2 predicts poor prognosis of patients with cervical cancer and promotes the proliferation and invasion of cervical cancer cells by regu
4 Aberrant expression of novel and previously described cell membrane markers in human breast cancer cell lines and tumors. Huang H, Groth J, Sossey-Alaou
5 Elevated expressions of MMP7, TROP2, and survivin are associated with survival, disease recurrence, and liver metastasis of colon cancer. Fang YJ, Lu ZH,
6 High expression of TROP2 correlates with poor prognosis in pancreatic cancer. Fong D, Moser P, Krammel C, Gostner JM, Margreiter R, Mitterer M, Gastl
7 The tetraspanin superfamily: molecular facilitators. Maecker HT, Todd SC, Levy S. FASEB J. 1997 May;11(6):428-42. Review.
8 Tetraspanin functions and associated microdomains. Hemler ME. Nat Rev Mol Cell Biol. 2005 Oct;6(10):801-11. Review.
9 Membrane microdomains and proteomics: lessons from tetraspanin microdomains and comparison with lipid rafts. Le Naour F, André M, Boucheix C, Rubins
10 Up-regulation of bone marrow stromal protein 2 (BST2) in breast cancer with bone metastasis. Cai D, Cao J, Li Z, Zheng X, Yao Y, Li W, Yuan Z. BMC Ca
11 Generation of potent antitumor CTL from patients with multiple myeloma directed against HM1.24. Rew SB, Peggs K, Sanjuan I, Pizzey AR, Koishihara Y,
12 Plasma membrane proteomics identifies bone marrow stromal antigen 2 as a potential therapeutic target in endometrial cancer. Yokoyama T, Enomoto T, Sera
13 Immunotherapy of multiple myeloma with a monoclonal antibody directed against a plasma cell-specific antigen, HM1.24. Ozaki S, Kosaka M, Wakatsuki S,
14 DNA methylation biomarkers for blood-based colorectal cancer screening. Lofton-Day C, Model F, Devos T, Tetzner R, Distler J, Schuster M, Song X, Lesc
15 Distribution and significance of nerve growth factor receptor (NGFR/p75NTR) in normal, benign and malignant breast tissue. Reis-Filho JS, Steele D, Di Palr
16 trk and ret proto-oncogene expression in human neuroblastoma specimens: high frequency of trk expression in non-advanced stages. Borrello MG, Bongarzon
17 A survey of immunohistochemical biomarkers for basal-like breast cancer against a gene expression profile gold standard. Won JR, Gao D, Chow C, Cheng J
18 Patterns of expression and function of the p75(NGFR) protein in pancreatic cancer cells and tumours. Wang W, Zhao H, Zhang S, Kang E, Chen Y, Ni C, Zh
19 Fibroblast growth factor 9 has oncogenic activity and is a downstream target of Wnt signaling in ovarian endometrioid adenocarcinomas. Hendrix ND, Wu R,
20 Expression and growth stimulatory effect of fibroblast growth factor 9 in human brain tumors. Todo T, Kondo T, Kirino T, Asai A, Adams EF, Nakamura S,
21 Expression of FGF and FGF receptor genes in human breast cancer. Penault-Llorca F, Bertucci F, Adelaide J, Parc P, Coulier F, Jacquemier J, Birnbaum D, d
22 Aryl hydrocarbon receptor activation and overexpression upregulated fibroblast growth factor-9 in human lung adenocarcinomas. Wang CK, Chang H, Chen
23 miR-26a suppresses tumor growth and metastasis by targeting FGF9 in gastric cancer. Deng M, Tang HL, Lu XH, Liu MY, Lu XM, Gu YX, Liu JF, He ZM.
24 Involvement of drug transporters in the synergistic action of FOLFOX combination chemotherapy. Theile D, Grebhardt S, Haefeli WE, Weiss J. Biochem Pha
25 Expression profiling of ABC transporters in a drug-resistant breast cancer cell line using AmpArray. Liu Y, Peng H, Zhang JT. Mol Pharmacol. 2005 Aug;68(
26 ABCC3 as a marker for multidrug resistance in non-small cell lung cancer. Zhao Y, Lu H, Yan A, Yang Y, Meng Q, Sun L, Pang H, Li C, Dong X, Cai L. Sc
27 Gene dosage alterations revealed by cDNA microarray analysis in cervical cancer: identification of candidate amplified and overexpressed genes. Narayan G,
28 ABCC drug efflux pumps and organic anion uptake transporters in human gliomas and the blood-tumor barrier. Bronger H, Konig J, Kopplow K, Steiner HH,
29 Colon tumour secretopeptidome: insights into endogenous proteolytic cleavage events in the colon tumour microenvironment. Greening DW, Kapp EA, Ji H,
30 Search for epithelial-specific mRNAs in peripheral blood of patients with colon cancer by RT-PCR. Solmi R, De Sanctis P, Zucchini C, Ugolini G, Rosati G,
31 EpCAM and gpA33 are markers of Barrett's metaplasia. Wong NA, Warren BF, Piris J, Maynard N, Marshall R, Bodmer WF. J Clin Pathol. 2006 Mar;59(3)
32 KLF4-dependent, PPARgamma-induced expression of GPA33 in colon cancer cell lines. Rageul J, Mottier S, Jarry A, Shah Y, Theoleyre S, Masson D, Gonz
33 A novel A33 promoter-based conditionally replicative adenovirus suppresses tumor growth and eradicates hepatic metastases in human colon cancer models.
34 Identification of the protein 4.1 binding interface on glycophorin C and p55, a homologue of the Drosophila discs-large tumor suppressor protein. Marfatia SM
35 Complete genomic organization of the human erythroid p55 gene (MPP1), a membrane-associated guanylate kinase homologue. Kim AC, Metzenberg AB, Sa
36 Molecular identification of a major palmitoylated erythrocyte membrane protein containing the src homology 3 motif. Ruff P, Speicher DW, Husain-Chishti A
37 The PDZ domain of human erythrocyte p55 mediates its binding to the cytoplasmic carboxyl terminus of glycophorin C. Analysis of the binding interface by i
38 PCDH24-induced contact inhibition involves downregulation of beta-catenin signaling. Ose R, Yanagawa T, Ikeda S, Ohara O, Koga H. Mol Oncol. 2009 Fel
39 ARMS depletion facilitates UV irradiation induced apoptotic cell death in melanoma. Liao YH, Hsu SM, Huang PH. Cancer Res. 2007 Dec 15;67(24):11547

40 Up-regulation of Flotillin-2 is associated with melanoma progression and modulates expression of the thrombin receptor protease activated receptor 1. Hazaril
41 High flotillin-2 expression is associated with lymph node metastasis and Breslow depth in melanoma. Doherty SD, Prieto VG, George S, Hazarika P, Duvic M
42 Identification of differentially expressed genes in metastatic and non-metastatic nasopharyngeal carcinoma cells by suppression subtractive hybridization. Yan
43 Flotillin-2 is associated with breast cancer progression and poor survival outcomes. Wang X, Yang Q, Guo L, Li XH, Zhao XH, Song LB, Lin HX. J Transl M
44 Trefoil factor-3 expression in human colon cancer liver metastasis. Babyatsky M, Lin J, Yio X, Chen A, Zhang JY, Zheng Y, Twyman C, Bao X, Schwartz M
45 Expression of trefoil peptides (TFF1, TFF2, and TFF3) in gastric carcinomas, intestinal metaplasia, and non-neoplastic gastric tissues. Leung WK, Yu J, Chan
46 ntestinal trefoil factor (TFF 3) and pS2 (TFF 1), but not spasmolytic polypeptide (TFF 2) mRNAs are co-expressed in normal, hyperplastic, and neoplastic hu
47 A comparative analysis by SAGE of gene expression profiles of esophageal adenocarcinoma and esophageal squamous cell carcinoma. van Baal JW, Milana
48 Plasma levels of trefoil factors are increased in patients with advanced prostate cancer. Vestergaard EM, Borre M, Poulsen SS, Nexo E, Torring N. Clin Canc
49 Minichromosome maintenance protein 2 (MCM2) is a stronger discriminator of increased proliferation in mucosa adjacent to colorectal cancer than Ki-67. Ha
50 Prognostic significance of metallothionein expression in correlation with Ki-67 expression in adenocarcinomas of large intestine. Dziegiel P, Forgacz J, Suder
51 Prognostic value of thymidylate synthase, Ki-67, and p53 in patients with Dukes' B and C colon cancer: a National Cancer Institute-National Surgical Adjuva
52 Immunohistochemical expression of p53, Bcl-2, and Ki-67 proteins in traditional serrated adenomas of colon. Jung JH, Kwon HJ, Kim TJ, Cho HJ, Kim HK,
53 Analysis of differential gene expression in colorectal cancer and stroma using fluorescence-activated cell sorting purification. Smith MJ, Culhane AC, Donova
54 Combination of strong MMP-2 and weak TIMP-2 immunostainings is a significant prognostic factor in endometrial carcinoma. Honkavuori-Toivola M, Santa
55 Expression of matrix metalloproteinases 1, 2, 9 and their tissue inhibitors in stage II non-small cell lung cancer: implications for MMP inhibition therapy. Pritc
56 In situ detection of matrix metalloproteinase-2 (MMP2) and the metalloproteinase inhibitor TIMP2 transcripts in human primary hepatocellular carcinoma and
57 Expression of tight and adherens junction proteins in ulcerative colitis associated colorectal carcinoma: upregulation of claudin-1, claudin-3, claudin-4, and be
58 The claudin gene family: expression in normal and neoplastic tissues. Hewitt KJ, Agarwal R, Morin PJ. BMC Cancer. 2006 Jul 12;6:186.
59 Claudins upregulation in human colorectal cancer. de Oliveira SS, de Oliveira IM, De Souza W, Morgado-Diaz JA. FEBS Lett. 2005 Nov 7;579(27):6179-85
60 Claudin-3 gene silencing with siRNA suppresses ovarian tumor growth and metastasis. Huang YH, Bao Y, Peng W, Goldberg M, Love K, Bumcrot DA, Cole
61 Claudins 2, 3, 4, and 5 in Paget's disease and breast carcinoma. Soini Y. Hum Pathol. 2004 Dec;35(12):1531-6.
62 Immunohistochemical profiles of claudin-3 in primary and metastatic prostatic adenocarcinoma. Bartholow TL, Chandran UR, Becich MJ, Parwani AV. Diag
63 Expression of metallothionein-3 in esophageal cancer and its correlations to expression of metallothionein-1 and -2. Tian ZQ, Wang XL, Wu W. Ai Zheng. 2
64 Hypermethylation of metallothionein-3 CpG island in gastric carcinoma. Deng D, El-Rifai W, Ji J, Zhu B, Trampont P, Li J, Smith MF, Powel SM. Carcinoge
65 Expression of MT-3 mRNA in human kidney, proximal tubule cell cultures, and renal cell carcinoma. Hoey JG, Garrett SH, Sens MA, Todd JH, Sens DA. To
66 Metallothionein 3: an androgen-upregulated gene enhances cell invasion and tumorigenesis of prostate carcinoma cells. Juang HH1, Chung LC, Sung HC, Fen
67 Differential gene expression of Eph receptors and ephrins in benign human tissues and cancers. Hafner C, Schmitz G, Meyer S, Bataille F, Hau P, Langmann
68 DNA-based assay for EPHB6 expression in breast carcinoma cells as a potential diagnostic test for detecting tumor cells in circulation. Fox BP, Kandpal RP.
69 Identification of EphB6 variant-derived epitope peptides recognized by cytotoxic T-lymphocytes from HLA-A24+ malignant glioma patients. Jin M, Komohar
70 High-throughput analysis of genome-wide receptor tyrosine kinase expression in human cancers identifies potential novel drug targets. Muller-Tidow C, Schw
71 EphB6 receptor significantly alters invasiveness and other phenotypic characteristics of human breast carcinoma cells. Fox BP, Kandpal RP. Oncogene. 2009
72 Expression of human alpha(1,3)fucosyltransferase antisense sequences inhibits selectin-mediated adhesion and liver metastasis of colon carcinoma cells. Wes
73 Different expression levels of alpha3/4 fucosyltransferases and Lewis determinants in ovarian carcinoma tissues and cell lines. Escrevente C, Machado E, Brit
74 Role of fucosyltransferases in the association between apomucin and Lewis antigen expression in normal and malignant gastric epithelium. Lopez-Ferrer A, de
75 Molecular cloning of MINK, a novel member of mammalian GCK family kinases, which is up-regulated during postnatal mouse cerebral development. Dan I,
76 Identification and functional characterization of a novel human misshapen/Nck interacting kinase-related kinase, hMINK beta. Hu Y, Leo C, Yu S, Huang BC
77 Mink1 regulates β-catenin-independent Wnt signaling via Prickle phosphorylation. Daulat AM, Luu O, Sing A, Zhang L, Wrana JL, McNeill H, Winklbauer R
78 Transcript analyses of stromal cell derived factors (SDFs): SDF-2, SDF-4 and SDF-5 reveal a different pattern of expression and prognostic association in hu
79 Genomic and transcriptomic prognostic factors in R0 Dukes B and C colorectal cancer patients. Vendrell E, Ribas M, Valls J, Sole X, Grau M, Moreno V, C
80 Increased expression of perforin and granzyme B genes in patients with metastatic melanoma treated with recombinant interleukin-2. Leger-Ravet MB, Mathi
81 Long-range epigenetic silencing at 2q14.2 affects most human colorectal cancers and may have application as a non-invasive biomarker of disease. Mayor R,
82 Inhibin/activin subunits (inhibin-alpha, -betaA and -betaB) are differentially expressed in human breast cancer and their metastasis. Mylonas I, Jeschke U, Sha
83 Current smoking-specific gene expression signature in normal bronchial epithelium is enhanced in squamous cell lung cancer. Boelens MC, van den Berg A,
84 Inhibin beta B: a useful tumor marker in uterine endometrioid adenocarcinomas? Mylonas I. Histol Histopathol. 2011 Nov;26(11):1415-22.
85 Indirect activation of blood coagulation in colon cancer. Wojtukiewicz MZ, Zacharski LR, Memoli VA, Kisiel W, Kudryk BJ, Rousseau SM, Stump DC. Thr
86 Differential expression of angiogenesis associated genes in prostate cancer bone, liver and lymph node metastases. Morrissey C, True LD, Roudier MP, Coler
87 Hyaluronan-CD44 interaction with IQGAP1 promotes Cdc42 and ERK signaling, leading to actin binding, Elk-1/estrogen receptor transcriptional activation,
88 Clinicopathologic significance of KIAA1199 overexpression in human gastric cancer. Matsuzaki S, Tanaka F, Mimori K, Tahara K, Inoue H, Mori M. Ann Su
89 Early insights into the function of KIAA1199, a markedly overexpressed protein in human colorectal tumors. Tiwari A, Schneider M, Fiorino A, Haider R, O
90 Transcriptional and epigenetic regulation of KIAA1199 gene expression in human breast cancer. Kuscu C, Evensen N, Kim D, Hu YJ, Zucker S, Cao J. PLoS
91 The calcineurin-binding protein cain is a negative regulator of synaptic vesicle endocytosis. Lai MM, Luo HR, Burnett PE, Hong JJ, Snyder SH. J Biol Chem.
92 Gamma subunit of the AP-1 adaptor complex binds clathrin: implications for cooperative binding in coated vesicle assembly. Doray B, Kornfeld S. Mol Biol
93 ARH cooperates with AP-1B in the exocytosis of LDLR in polarized epithelial cells. Kang RS, Fölsch H. J Cell Biol. 2011 Apr 4;193(1):51-60. doi: 10.1083/
94 EpsinR: an AP1/clathrin interacting protein involved in vesicle trafficking. Mills IG, Praefcke GJ, Vallis Y, Peter BJ, Olesen LE, Gallop JL, Butler PJ, Evans
95 Colon cancer secreted protein-2 (CCSP-2), a novel candidate serological marker of colon neoplasia. Xin B, Platzer P, Fink SP, Reese L, Nosrati A, Willson J
96 Pancreatic Expression of DOG1: A Novel Gastrointestinal Stromal Tumor (GIST) Biomarker. Ardeleanu C, Arsene D, Hinescu M, Andrei F, Gutu D, Luca L
97 A novel monoclonal antibody against DOG1 is a sensitive and specific marker for gastrointestinal stromal tumors. Espinosa I, Lee CH, Kim MK, Rouse BT,
98 TMEM16A alternative splicing coordination in breast cancer. Ubby I, Bussani E, Colonna A, Stacul G, Locatelli M, Scudieri P, Galietta L, Pagani F. Mol Ca
99 Ubiquitin-ribosomal protein S27a gene overexpressed in human colorectal carcinoma is an early growth response gene. Wong JM, Mafune K, Yow H, Rivers
100 Metallopanstimulin as a novel tumor marker in sera of patients with various types of common cancers: implications for prevention and therapy. Fernandez-Pol
101 Identification of metallopanstimulin-1 as a member of a tumor associated antigen in patients with breast cancer. Atsuta Y, Aoki N, Sato K, Oikawa K, Nochi
102 In vitro and in vivo evidence of metallopanstimulin-1 in gastric cancer progression and tumorigenicity. Wang YW, Qu Y, Li JF, Chen XH, Liu BY, Gu QL, Z
103 Genes for human general transcription initiation factors TFIIIB, TFIIIB-associated proteins, TFIIIC2 and PTF/SNAPC: functional and positional candidates fo
104 RNA polymerase III transcription factor TFIIIC2 is overexpressed in ovarian tumors. Winter AG, Sourvinos G, Allison SJ, Tosh K, Scott PH, Spandidos DA,
105 Epigenetic silencing of a Ca(2+)-regulated Ras GTPase-activating protein RASAL defines a new mechanism of Ras activation in human cancers. Jin H, Wang
106 Gene expression profiling in hepatocellular carcinoma: upregulation of genes in amplified chromosome regions. Skawran B, Steinemann D, Weigmann A, Fle
107 IQGAPs in cancer: a family of scaffold proteins underlying tumorigenesis. White CD, Brown MD, Sacks DB. FEBS Lett. 2009 Jun 18;583(12):1817-24.
108 Serological identification and bioinformatics analysis of immunogenic antigens in multiple myeloma. Zhou FL, Zhang WG, Chen G, Zhao WH, Cao XM, Che
109 Calpain5 expression is decreased in endometriosis and regulated by HOXA10 in human endometrial cells. Penna I, Du H, Ferriani R, Taylor HS. Mol Hum R
110 Significance of the expression of proliferation-associated nucleolar antigen p120 in human colorectal tumors. Ueki T, Nakayama Y, Sugao Y, Kohno K, Matsu
111 Altered transcription control is responsible for the increased level of proliferation-associated P120 in rapidly growing breast carcinoma. Fonagy A, Swiderski
112 Prognostic value of nucleolar protein p120 in patients with resected lung adenocarcinoma. Sato G, Saijo Y, Uchiyama B, Kumano N, Sugawara S, Fujimura S
113 Differential gene expression profile reveals deregulation of pregnancy specific beta1 glycoprotein 9 early during colorectal carcinogenesis. Salahshor S, Gonc
114 Oncofetal markers CA 19-9, CA 125 and SP1 in healthy children and in children with malignancy. Heikinheimo M, Rajantie J, Kuusela P, Kallio MJ, Siimes
115 Prognostic factors in breast cancer: immunohistochemical staining for SP1 and NCRC 11 related to survival, tumour epidermal growth factor receptor and oes
116 Molecular and functional characterization of choline transporter in human colon carcinoma HT-29 cells. Kouji H, Inazu M, Yamada T, Tajima H, Aoki T, Ma
117 Phosphoglycerate mutase, 2,3-bisphosphoglycerate phosphatase and enolase activity and isoenzymes in lung, colon and liver carcinomas. Durany N, Joseph J
118 Over-expression of gastrin-releasing peptide in human esophageal squamous cell carcinomas. Fang MZ, Liu C, Song Y, Yang GY, Nie Y, Liao J, Zhao X, Sh
119 Endocrine fibroblast growth factor FGF19 promotes prostate cancer progression. Feng S, Dakhova O, Creighton CJ, Ittmann M. Cancer Res. 2013 Apr 15;73
120 Targeting FGF19 inhibits tumor growth in colon cancer xenograft and FGF19 transgenic hepatocellular carcinoma models. Desnoyers LR, Pai R, Ferrando R
121 Silencing of Peroxiredoxin 2 and aberrant methylation of 33 CpG islands in putative promoter regions in human malignant melanomas. Furuta J, Nobeyama Y

122 MFng is dispensable for mouse pancreas development and function. Svensson P, Bergqvist I, Norlin S, Edlund H. Mol Cell Biol. 2009 Apr;29(8):2129-38.

Criterion 6
Yes
Yes
Yes
Yes
No
Yes
Yes
Yes
Yes
Yes
Yes
Yes
Yes
Yes
Yes
Yes
No
No
Yes
Yes
Yes
Yes
Yes
Yes
Yes
Yes
Yes
Yes
Yes
Yes
Yes
Yes
No
Yes
No
Yes
No
Yes
Yes
No
No
Yes
Yes
Yes
Yes
No
No
No
Yes
Yes

Yes
Yes
Yes
No
No
Yes
No
Yes
Yes
Yes
No
Yes
Yes
Yes
Yes
Yes

Ther. 2008 Feb;7(2):280-5.
15;12(10):3057-63.
ulating ERK signaling pathway. Liu T, Liu Y, Bao X, Tian J, Liu Y, Yang X. PLoS One. 2013 Sep 27;8(9):e75864.
i K, Hawthorn L, Beall S, Geradts J. Clin Cancer Res. 2005 Jun 15;11(12):4357-64.
Wang GQ, Pan ZZ, Zhou ZW, Yun JP, Zhang MF, Wan DS. Int J Colorectal Dis. 2009 Aug;24(8):875-84. Epub 2009 May 7.
G, Spizzo G. Br J Cancer. 2008 Oct 21;99(8):1290-5. Epub 2008 Sep 23.

stein E. Proteomics. 2006 Dec;6(24):6447-54.
ncer. 2009 Apr 1;9:102.
Kawai S, Kosaka M, Ozaki S, Chain B, Yong KL. Clin Cancer Res. 2005 May 1;11(9):3377-84.
ada S, Morimoto A, Matsuzaki S, Ueda Y, Yoshino K, Fujita M, Kyo S, Iwahori K, Fujimoto M, Kimura T, Naka T. Int J Cancer. 2013 Jan 15;132(2):472-84
Abe M, Koishihara Y, Matsumoto T. Blood. 1997 Oct 15;90(8):3179-86.
he R, Liebenberg V, Ebert M, Molnar B, Grutzmann R, Pilarsky C, Sledziewski A. Clin Chem. 2008 Feb;54(2):414-23. Epub 2007 Dec 18.
na S, Jones RL, Savage K, James M, Milanezi F, Schmitt FC, Ashworth A. Mod Pathol. 2006 Feb;19(2):307-19.
e I, Pierotti MA, Luksch R, Gasparini M, Collini P, Pilotti S, Rizzetti MG, Mondellini P, De Bernardi B, et al. Int J Cancer. 1993 Jun 19;54(4):540-5.
, Lau SY, Ellis MJ, Perou CM, Bernard PS, Nielsen TO. Mod Pathol. 2013 Nov;26(11):1438-50
ang S, Zhu M. Eur J Surg Oncol. 2009 Aug;35(8):826-32. Epub 2008 Nov 28.
Kuick R, Schwartz DR, Fearon ER, Cho KR. Cancer Res. 2006 Feb 1;66(3):1354-62.
keda K, Kurokawa T. Neurosurgery. 1998 Aug;43(2):337-46.
eLapeyriere O. Int J Cancer. 1995 Apr 10;61(2):170-6.
PH, Chang JT, Kuo YC, Ko JL, Lin P. Int J Cancer. 2009 Aug 15;125(4):807-15.
PLoS One. 2013 Aug 28;8(8):e72662.
rmacol. 2009 Jul 19.
2):430-8. Epub 2005 May 18.
i Rep. 2013 Nov 1;3:3120.
Bourdon V, Chaganti S, Arias-Pulido H, Nandula SV, Rao PH, Gissmann L, Durst M, Schneider A, Pothuri B, Mansukhani M, Basso K, Chaganti RS, Murty VV. Genes
Ahmadi R, Herold-Mende C, Keppler D, Nies AT. Cancer Res. 2005 Dec 15;65(24):11419-28.
Speed TP, Simpson RJ. Biochim Biophys Acta. 2013 Nov;1834(11):2396-407.
Del Governatore M, Coppola D, Yeatman TJ, Lenzi L, Caira A, Zanotti S, Taffurelli M, Carinci P, Valvassori L, Strippoli P. Int J Oncol. 2004 Oct;25(4):1049-56.
:260-3. Epub 2006 Feb 10.
alez FJ, Laboisse CL, Denis MG. Int J Cancer. 2009 Jun 23.
Cafferata EG, Maccio DR, Lopez MV, Viale DL, Carbone C, Mazzolini G, Podhajcer OL. Clin Cancer Res. 2009 May 1;15(9):3037-49. Epub 2009 Mar 31.
M, Leu RA, Branton D, Chishti AH. J Biol Chem. 1995 Jan 13;270(2):715-9.
ahr KE, Marfatia SM, Chishti AH. Genomics. 1996 Jan 15;31(2):223-9.
. Proc Natl Acad Sci U S A. 1991 Aug 1;88(15):6595-9.
n vitro mutagenesis. Marfatia SM, Morais-Cabral JH, Kim AC, Byron O, Chishti AH. J Biol Chem. 1997 Sep 26;272(39):24191-7.
b;3(1):54-66. Epub 2008 Nov 6.
-56.

ka P, McCarty MF, Prieto VG, George S, Babu D, Koul D, Bar-Eli M, Duvic M. Cancer Res. 2004 Oct 15;64(20):7361-9.
M. Melanoma Res. 2006 Oct;16(5):461-3.
g XY, Ren CP, Wang L, Li H, Jiang CJ, Zhang HB, Zhao M, Yao KT. Cell Oncol. 2005;27(4):215-23.
Med. 2013 Aug 15;11:190.
I, Thung S, Lawrence Werther J, Itzkowitz S. Clin Exp Metastasis. 2009;26(2):143-51. Epub 2008 Nov 2.
FK, To KF, Chan MW, Ebert MP, Ng EK, Chung SC, Malfertheiner P, Sung JJ. J Pathol. 2002 Aug;197(5):582-8.
man breast epithelium. Poulsom R, Hanby AM, Lalani EN, Hauser F, Hoffmann W, Stamp GW. J Pathol. 1997 Sep;183(1):30-8.
F, Rygiel AM, Sondermeijer CM, Spek CA, Bergman JJ, Peppelenbosch MP, Krishnadath KK. Cell Oncol. 2008;30(1):63-75.
er Res. 2006 Feb 1;12(3 Pt 1):807-12.
nna-Morris A, Badvie S, Cohen P, McCullough T, Andreyev HJ, Allen-Mersh TG. J Clin Pathol. 2009 Apr;62(4):325-30. Epub 2008 May 12.
E, Surowiak P, Kornafel J, Zabel M. Histol Histopathol. 2003 Apr;18(2):401-7.
nt Breast and Bowel Project collaborative study. Allegra CJ, Paik S, Colangelo LH, Parr AL, Kirsch I, Kim G, Klein P, Johnston PG, Wolmark N, Wieand HS. J Clin Onc
Cheung DY, Kim JI, Kim JK. Korean J Gastroenterol. 2013 Dec;62(6):336-43.
n M, Coffey JC, Barry BD, Kelly MA, Higgins DG, Wang JH, Kirwan WO, Cotter TG, Redmond HP. Br J Cancer. 2009 May 5;100(9):1452-64
la M, Soini Y, Turpeenniemi-Hujanen T, Talvensaari-Mattila A. Dis Markers. 2013;35(4):261-6.
chard SC, Nicolson MC, Lloret C, McKay JA, Ross VG, Kerr KM, Murray GI, McLeod HL. Oncol Rep. 2001 Mar-Apr;8(2):421-4.
l in liver metastasis. Musso O, Theret N, Campion JP, Turlin B, Milani S, Grappone C, Clement B. J Hepatol. 1997 Mar;26(3):593-605.
ta-catenin. Mees ST, Mennigen R, Spieker T, Rijcken E, Senninger N, Haier J, Bruewer M. Int J Colorectal Dis. 2009 Apr;24(4):361-8. Epub 2009 Jan 29.

. Epub 2005 Oct 13.
G, Langer R, Anderson DG, Sawicki JA. Proc Natl Acad Sci U S A. 2009 Mar 3;106(9):3426-30. Epub 2009 Feb 10.

n Pathol. 2011 Jan 21;6:12.
)08 Feb;27(2):160-4. Chinese.
enesis. 2003 Jan;24(1):25-9.
oxicol Lett. 1997 Jul 21;92(2):149-60.
g TH, Lee YH, Chang PL, Tsui KH. Prostate. 2013 Oct;73(14):1495-506.
T, Dietmaier W, Landthaler M, Vogt T. Clin Chem. 2004 Mar;50(3):490-9. Epub 2004 Jan 15.
Cancer Genomics Proteomics. 2010 Jan-Feb;7(1):9-16.
ra Y, Shichijo S, Harada M, Yamanaka R, Miyamoto S, Nikawa J, Itoh K, Yamada A. Oncol Rep. 2008 May;19(5):1277-83.
wable J, Steffen B, Tidow N, Brandt B, Becker K, Schulze-Bahr E, Halfter H, Vogt U, Metzger R, Schneider PM, Buchner T, Brandts C, Berdel WE, Serve H. Clin Cancer
Apr 9;28(14):1706-13. Epub 2009 Feb 23.
ton BW, Hiller KM, Mayben JP, Manousos GA, Bendt KM, Liu R, Cusack JC Jr. Cancer Res. 1999 May 1;59(9):2127-35.
to C, Reis CA, Stoeck A, Runz S, Marme A, Altevogt P, Costa J. Int J Oncol. 2006 Sep;29(3):557-66.
e Bolos C, Barranco C, Garrido M, Isern J, Carlstedt I, Reis CA, Torrado J, Real FX. Gut. 2000 Sep;47(3):349-56.
Watanabe NM, Kobayashi T, Yamashita-Suzuki K, Fukagaya Y, Kajikawa E, Kimura WK, Nakashima TM, Matsumoto K, Ninomiya-Tsuji J, Kusumi A. FEBS Lett. 200
, Wang H, Shen M, Luo Y, Daniel-Issakani S, Payan DG, Xu X. J Biol Chem. 2004 Dec 24;279(52):54387-97
R, Angers S. Mol Cell Biol. 2012 Jan;32(1):173-85.
man breast cancer. Kang H, Escudero-Esparza A, Douglas-Jones A, Mansel RE, Jiang WG. Int J Oncol. 2009 Jul;35(1):205-11.
apella G, Peinado MA. Int J Oncol. 2007 May;30(5):1099-107.
ot C, Portier A, Brandely M, Galanaud P, Fridman WH, Emilie D. Cancer Immunol Immunother. 1994 Jul;39(1):53-8.
Casadome L, Azuara D, Moreno V, Clark SJ, Capella G, Peinado MA. Br J Cancer. 2009 May 19;100(10):1534-9. Epub 2009 Apr 21.
abani N, Kuhn C, Friese K, Gerber B. Oncol Rep. 2005 Jan;13(1):81-8.
Fehrmann RS, Geerlings M, de Jong WK, te Meerman GJ, Sietsma H, Timens W, Postma DS, Groen HJ. J Pathol. 2009 Jun;218(2):182-91.

omb Haemost. 1989 Dec 29;62(4):1062-6.
man IM, Hawley S, Nelson PS, Coleman R, Wang YC, Corey E, Lange PH, Higano CS, Vessella RL. Clin Exp Metastasis. 2008;25(4):377-88. Epub 2007 Oct 31.
and ovarian cancer progression. Bourguignon LY, Gilad E, Rothman K, Peyrollier K. J Biol Chem. 2005 Mar 25;280(12):11961-72. Epub 2005 Jan 17.
urg Oncol. 2009 Jul;16(7):2042-51. Epub 2009 May 12.
koniewski MJ, Roschitzki B, Uzozie A, Menigatti M, Jiricny J, Marra G. PLoS One. 2013 Jul 23;8(7):e69473
One. 2012;7(9):e44661.
2000 Nov 3;275(44):34017-20.
Cell. 2001 Jul;12(7):1925-35.
/jcb.201012121. Epub 2011 Mar 28.
PR, McMahon HT. J Cell Biol. 2003 Jan 20;160(2):213-22. Epub 2003 Jan 21.
K, Wilson K, Markowitz S. Oncogene. 2005 Jan 20;24(4):724-31.
, Popescu LM. Appl Immunohistochem Mol Morphol. 2009 May 4.
Subramanian S, Montgomery K, Varma S, Corless CL, Heinrich MC, Smith KS, Wang Z, Rubin B, Nielsen TO, Seitz RS, Ross DT, West RB, Cleary ML, van de Rijn M.
ncer. 2013 Jul 16;12:75.
EN, Ravikumar TS, Steele GD Jr, Chen LB. Cancer Res. 1993 Apr 15;53(8):1916-20.
JA. Anticancer Res. 1996 Jul-Aug;16(4B):2177-85.
H, Miyokawa N, Hirata S, Kimura S, Sasajima T, Katagiri M. Cancer Lett. 2002 Aug 8;182(1):101-7.
hu ZG. Clin Cancer Res. 2006 Aug 15;12(16):4965-73.
or tumour predisposition or inherited genetic diseases? Purrello M, Di Pietro C, Rapisarda A, Amico V, Giunta V, Engel H, Stevens S, Hsieh Y, Teichman M, Wang Z, Sic
, White RJ. Proc Natl Acad Sci U S A. 2000 Nov 7;97(23):12619-24.
g X, Ying J, Wong AH, Cui Y, Srivastava G, Shen ZY, Li EM, Zhang Q, Jin J, Kupzig S, Chan AT, Cullen PJ, Tao Q. Proc Natl Acad Sci U S A. 2007 Jul 24;104(30):123
mming P, Becker T, Flik J, Kreipe H, Schlegelberger B, Wilkens L. Mod Pathol. 2008 May;21(5):505-16.

n YX, Tian W, Liu J, Liu SH. Cancer Immunol Immunother. 2006 Aug;55(8):910-7.
eprod. 2008 Oct;14(10):613-8.
o K, Sugimoto Y, Yamada Y, Kuwano M, Tsuneyoshi M. Hum Pathol. 1997 Jan;28(1):74-9.
C, Freeman JW. Int J Cancer. 1995 Jan 27;60(3):407-12.
, Sato M, Sagawa M, Ohkuda K, Koike K, Minami Y, Satoh K, Nukiwa T. J Clin Oncol. 1999 Sep;17(9):2721-7.
alves J, Chetty R, Gallinger S, Woodgett JR. BMC Cancer. 2005 Jun 27;5:66.
MA. Br J Cancer. 1990 Nov;62(5):865-7.
strogen receptor status. Wright C, Angus B, Napier J, Wetherall M, Udagawa Y, Sainsbury JR, Johnston S, Carpenter F, Horne CH. J Pathol. 1987 Dec;153(4):325-31.
tsumiya T. Arch Biochem Biophys. 2009 Mar 1;483(1):90-8.
, Campo E, Molina R, Carreras J. Br J Cancer. 1997;75(7):969-77.
imada Y, Wang LD, Yang CS. Carcinogenesis. 2004 Jun;25(6):865-71.
(8):2551-62.
, Hötzel K, Le T, Ross J, Carano R, D'Souza A, Qing J, Mohtashemi I, Ashkenazi A, French DM. Oncogene. 2008 Jan 3;27(1):85-97.
, Umebayashi Y, Otsuka F, Kikuchi K, Ushijima T. Cancer Res. 2006 Jun 15;66(12):6080-6.

Chromosomes Cancer. 2007 Apr;46(4):373-84.

ɔl. 2003 Jan 15;21(2):241-50.

· Res. 2004 Feb 15;10(4):1241-9.

0 Mar 3;469(1):19-23.

Am J Surg Pathol. 2008 Feb;32(2):210-8.

hel G, Roeder R, Grzeschik KH. Oncogene. 2001 Aug 9;20(35):4877-83.

353-8.

Supplemental Table 2. Characteristics of all study subjects used in this study.

**Characteristics**	**Used for ELISA**				**Used for IHC analysis CRC case**
	**1st phase**		**2nd phase**		
	**CRC Case**	**Control**	**CRC Case**	**Control**	
**Age (yr, median ±SEM)**	67.5 ± 12.3	43.1 ± 12.7	65.3 ± 12.7	45.3 ± 10.1	62.9 ± 12.8
**Gender**-Male [N, (%)]	22(68.8)	15(46.9)	73(60.8)	61(50.8)	74(56.1)
Female	10(31.2)	17(53.1)	47(39.2)	59(49.2)	58(43.9)
**Location (Organ Code)**					
Colon			62(51.7)		49(37.1)
Rectum			46(38.3)		70(53.0)
Others			12(10.0)		13(9.9)
**Histological grade**					
Well differentiation			10(8.3)		25(18.9)
Moderate differentiation			99(82.5)		96(72.7)
Poor differentiation			10(8.3)		9(6.8)
Unknown			1(0.8)		2(1.5)
**Histological type**					
Adenocarcinoma			115(95.8)		119(90.2)
Mucinous carcinoma			4(3.3)		10(7.6)
Others			1(0.8)		3(2.3)
**TNM stage**					
0/I/II			61(50.8)		67(60.8)
III/IV			59(49.2)		65(49.2)
**CEA level**					
＜ 5 ng/ml			93(77.5)		63(47.7)
≧ 5 ng/ml			27(22.5)		64(48.5)
Unknown			0(0)		5(3.8)
**Total case No.**	32	32	120	120	132

Supplemental Table 3. Association of BST2 plasma levels with different clinicopathologic characteristics of the 120 CRC patients.

Characteristics	Number	BST2 (ng/ml)	*p*-value[a]
**Gender**			
Male	73	2.40±1.66	0.63
Female	47	2.26±1.21	
**Age (years)**			
＜ 65	54	2.03±1.63	0.03
≧ 65	66	2.61±1.34	
**Location (Organ Code)**			
Colon	62	2.54±1.67	0.70
Rectum	46	2.12±1.30	
**Histological grade**			
Well differentiation	10	2.86±1.69	
Moderate differentiation	99	2.25±1.48	0.39
Poor differentiation	10	2.86±1.46	
**Histological type**			
Adenocarcinoma	115	2.28±1.48	0.05
Mucinous carcinoma	3	4.63±0.45	
**TNM stage**			
I/II	61	2.42±1.76	0.57
III/IV	59	2.27±1.18	
**Distant metastasis**			
No	106	2.33±1.55	0.83
Yes	14	2.42±1.06	
**CEA level**			
＜ 5 ng/ml	93	2.18±1.22	0.10
≧ 5 ng/ml	27	2.90±2.13	
**Albumin**			
＜ 3.5 g/dL	14	4.14±2.46	< 0.01
≧ 3.5 g/dL	105	2.12±1.14	
**Three years DFS[b]**			
＜ 3 years	86	2.50±1.67	0.02
≧ 3 years	34	1.95±0.81	
**Chemotherapy**			
No	56	2.41±1.84	0.88
Yes	64	2.36±1.17	

a: The *p*-values were determined by independent t test.
b: disease free survival

Supplemental Table 4. Association of BST2 IHC scores with different clinicopathologic characteristics of the 132 CRC patients.

Characteristics	Number	IHC score	p-value[a]
**Gender**			
Male	74	144.5±87.3	0.39
Female	58	137.7±90.1	
**Age (years)**			
＜ 65	59	147.8±85.2	0.15
≧ 65	73	136.5±90.4	
**Location (Organ Code)**			
Colon	49	145.5±98.6	0.47
Rectum	70	139.4±83.1	
**Histological grade**			
Well differentiation	25	144.4±99.7	
Moderate differentiation	96	139.1±88.2	0.60
Poor differentiation	9	152.2±56.1	
**Histological type**			
Adenocarcinoma	119	140.3±89.9	0.92
Mucinous carcinoma	9	138.8±68.2	
**TNM stage**			
I/II	67	133.7±81.1	0.05
III/IV	62	149.1±94.7	
**Distant metastasis**			
No	115	138.9±88.9	0.11
Yes	16	158.1±80.4	
**CEA level**			
＜ 5 ng/ml	63	135.1±94.4	0.07
≧ 5 ng/ml	64	149.3±82.7	
**Five years survival**			
＜ 5 years	59	155.9±86.7	< 0.01
≧ 5 years	73	130.0±83.7	
**Chemotherapy**			
No	47	151.1±86.9	0.06
Yes	77	135.3±89.1	

a: The p-values were determined by independent t test.

Fig. S1

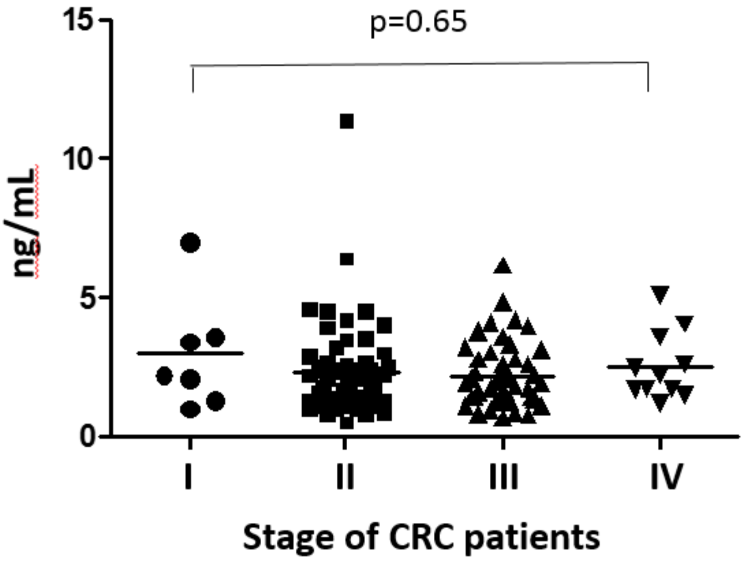

Supplemental Figure 1. The ELISA analysis of BST2 according to stages in CRC patients' plasma was shown. The BST2 serum level were 2.20±1.15 ng/mL (stage 1, n=7), 2.08±0.74 ng/mL (stage 2, n=56), 1.90±0.73 ng/mL (stage 3, n=46), and 2.20 ±0.96 ng/mL (stage 4, n=11). There was no statistical difference among all stages for CRC patients (p=0.65, ANOVA).
